# Nrf2 Is a Potential Modulator for Orchestrating Iron Homeostasis and Redox Balance in Cancer Cells

**DOI:** 10.3389/fcell.2021.728172

**Published:** 2021-09-13

**Authors:** Lingyan Zhang, Jian Zhang, Yuanqing Jin, Gang Yao, Hai Zhao, Penghai Qiao, Shuguang Wu

**Affiliations:** Institute of Laboratory Animal Science, Guizhou University of Traditional Chinese Medicine, Guiyang, China

**Keywords:** Nrf2, iron homeostasis, redox balance, cancer, ferroptosis

## Abstract

Iron is an essential trace mineral element in almost all living cells and organisms. However, cellular iron metabolism pathways are disturbed in most cancer cell types. Cancer cells have a high demand of iron. To maintain rapid growth and proliferation, cancer cells absorb large amounts of iron by altering expression of iron metabolism related proteins. However, iron can catalyze the production of reactive oxygen species (ROS) through Fenton reaction. Nuclear factor (erythroid-derived 2)-like 2 (Nrf2) is an important player in the resistance to oxidative damage by inducing the transcription of antioxidant genes. Aberrant activation of Nrf2 is observed in most cancer cell types. It has been revealed that the over-activation of Nrf2 promotes cell proliferation, suppresses cell apoptosis, enhances the self-renewal capability of cancer stem cells, and even increases the chemoresistance and radioresistance of cancer cells. Recently, several genes involving cellular iron homeostasis are identified under the control of Nrf2. Since cancer cells require amounts of iron and Nrf2 plays pivotal roles in oxidative defense and iron metabolism, it is highly probable that Nrf2 is a potential modulator orchestrating iron homeostasis and redox balance in cancer cells. In this hypothesis, we summarize the recent findings of the role of iron and Nrf2 in cancer cells and demonstrate how Nrf2 balances the oxidative stress induced by iron through regulating antioxidant enzymes and iron metabolism. This hypothesis provides new insights into the role of Nrf2 in cancer progression. Since ferroptosis is dependent on lipid peroxide and iron accumulation, Nrf2 inhibition may dramatically increase sensitivity to ferroptosis. The combination of Nrf2 inhibitors with ferroptosis inducers may exert greater efficacy on cancer therapy.

## Introduction

Iron is an essential trace mineral element in the body and is engaged in a wide range of biological processes, such as oxygen transport, electron transport, energy metabolism, DNA synthesis and repair, etc. (Torti et al., 2018). Cancer cells have a high demand for iron compared with normal cells to initiate carcinogenesis, sustain uncontrolled proliferation, and other events necessary for cancer progression. However, excess iron is toxic. Iron can catalyze the production of a highly toxic hydroxyl radical *via* Fenton/Haber–Weiss reaction cycle (Torti and Torti, 2020). Iron-mediated oxidative stress not only induces oxidative damage but also induces ferroptosis *via* lipid peroxidation (Anandhan et al., 2020; Chen et al., 2020). Considering the high levels of intracellular iron within cancer cells, ferroptosis induction provides an alternative strategy for cancer therapy.

Nuclear factor (erythroid-derived 2)-like 2 (Nrf2) is an important transcription regulator of cellular resistance to oxidative stress (Bellezza et al., 2018). Nrf2 is found to mediate transcription of a set of antioxidant and cellular protective genes, attenuating cellular injury from oxidative stress. Nrf2-mediated antioxidant capabilities have been demonstrated to prevent multiple diseases, including neurodegenerative diseases (Song and Long, 2020), cardiovascular disorders (Vashi and Patel, 2021), osteoporosis (Sun et al., 2015), and inflammation (Ahmed et al., 2017). Oxidative stress is suggested to be involved in cancer initiation and progression. To maintain redox balance and avoid oxidative damage, cancer cells upregulate their antioxidant capacity (Reczek and Chandel, 2017). Indeed, a variety of cancers exhibit hyperactivation of Nrf2, conferring aggressive proliferation (Okazaki et al., 2020), metastasis (Lignitto et al., 2019), chemoresistance (Jeong et al., 2020), and radioresistance (Wang Z. et al., 2020). Nrf2 hyperactivation counteracts the large amount of reactive oxygen species (ROS) production induced by excess iron (Wu et al., 2019; Zimta et al., 2019). On the other hand, ferritin (an iron storage protein) and ferroportin 1 (FPN1, an iron exporter protein) are under the control of Nrf2 (Kasai et al., 2018). Ferritin and FPN1 reduce the cellular free iron by enhancing iron sequestration and export (Chen et al., 2021). Since cancer cells have an enhanced iron uptake and Nrf2 plays pivotal roles in oxidative defense and iron metabolism, it is highly probable that Nrf2 is a potential modulator orchestrating iron homeostasis and redox balance in cancer cells. Oxidative stress can be induced by the high levels of iron accumulation in cancer cells and activates the Nrf2 pathway (Bellezza et al., 2018; Nakamura et al., 2019). Nrf2 not only eliminates ROS by inducing expression of antioxidant genes but also removes iron by FPN1 and neutralizes free iron by ferritin. From this point, Nrf2 exhibits pro-oncogenic activity (Jung et al., 2018). Nrf2 inhibition has attracted more attention on cancer therapy (Qin et al., 2019). However, most of the Nrf2 inhibitors exhibit low potency, limiting its clinical application (Jung et al., 2018; Qin et al., 2019). A combination of Nrf2 inhibitors with ferroptosis inducers may exert greater efficacy on cancer therapy.

## Iron, Oxidative Stress, and Ferroptosis

As a transition metal, iron in its free state is toxic to our body. Circulating ferric iron is bound by transferrin (Tf) and is taken up through transferrin receptor 1 (TfR1) mediated endocytosis. The ferric iron is then released from transferrin and is reduced to ferrous iron by ferrireductase six-transmembrane epithelial antigen of the prostate 3 (STEAP3) proteins in the endosomes. Subsequently, the ferrous iron enters the cytoplasm *via* divalent metal transporter 1 (DMT1) and is utilized for the synthesis of heme and iron-sulfur (Fe/S) clusters. The excess iron can be stored in ferritin, the major iron-storage protein at the cellular level, or is exported from cells through FPN1, the only known iron exporter (Sacco et al., 2021; Zhang et al., 2021). Hepcidin, an iron regulator synthesized in the liver, controls iron export from cells by inducing the internalization and degradation of FPN1 (Camaschella et al., 2020). Under normal condition, the cellular iron homeostasis is tightly regulated to avoid the enhanced labile iron pool (LIP), a pool of chelatable and redox-active iron (Nakamura et al., 2019). As a transition metal, iron can undergo redox cycling reactions between ferrous (Fe^2+^) and ferric (Fe^3+^) oxidation states. This redox activity enables ferrous iron to transfer an electron to hydrogen peroxide to generate highly toxic hydroxyl radical (HO•) and ferric iron (Nakamura et al., 2019). The reaction between ferrous iron and hydrogen peroxide to yield hydroxyl radicals is called Fenton reaction. In the presence of superoxide, the ferric iron can be reduced back to ferrous iron, which then undergoes Fenton reactions. Hydroxyl radicals are responsible for the cytotoxic effects by reacting with lipids, proteins, and nucleic acids (Nakamura et al., 2019).

**FIGURE 1:**
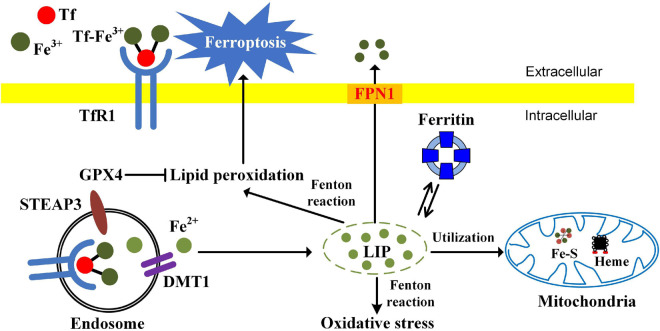
The relationships among iron, oxidative stress, and ferroptosis. TfR1-mediated iron uptake is the major way for iron acquisition by most mammalian cells. When Tf binds to the cell-surface TfR1, the complex is internalized by receptor-mediated endocytosis. Ferric iron is then released and reduced to the ferrous state by ferrireductase STEAP3. After that, Fe^2+^ is transported into the cytosol from the endosome by DMT1. In mitochondria, iron is utilized for the synthesis of Fe-S clusters and heme. Ferritin is the major iron-storage protein. Cellular free iron catalyzes the formation of ROS and causes oxidative stress *via* Fenton reaction. The excessive iron-dependent generation of lipid peroxidation can lead to ferroptosis.

Ferroptosis is a type of iron dependent oxidative cell death caused by the accumulation of ROS from the Fenton reaction and iron-mediated lipid peroxidation (Chen et al., 2020). Morphologically, cells undergoing ferroptosis exhibit unique characteristics, including reduced mitochondrial volume, increased bilayer membrane density, and reduction or disappearance of mitochondrial crista. Glutathione peroxidase 4 (GPX4), a kind of GSH-dependent reductase, plays a central role in regulating ferroptosis. GPX4 converts lipid hydroperoxides to lipid alcohols, preventing the formation of toxic lipid ROS. Inactivation of antioxidant GPX4 dependent systems provokes ferroptosis (Lei et al., 2021). Cellular iron is considered as an essential factor in ferroptosis. Increased intracellular ferrous iron levels are often observed during the induction of ferroptosis (Hou et al., 2016). Enhanced iron uptake markedly increases the sensitivity of cells to ferroptosis inducers and even directly induces ferroptosis (Gao et al., 2019). Iron chelators significantly inhibit the occurrence of ferroptosis (Chen et al., 2020). Ferroptosis opens new avenues for those chemoresistant and radioresistant cancers. The relationships among iron, oxidative stress, and ferroptosis are summarized in Figure 1.

## Iron and Cancer

### Epidemiological Studies

People with iron overload have a higher cancer risk (Torti et al., 2018). Many epidemiological studies have investigated the association between iron status and cancer risk. The risk of cancer occurrence and mortality in the group with above 60% transferrin saturation relative to the 0–30% group is 1.81 and 1.73 (Stevens et al., 1994). For participants with serum iron higher than 141 μg/dl, their relative risks of cancer mortality are 1.86 compared with those with serum iron of 61–94 μg/dl (Wu et al., 2004). For people with transferrin saturation more than 43%, dietary iron more than 18 mg/day increases cancer risk (Mainous et al., 2005). Women with serum ferritin higher than 160 μg/L may have an increased risk of cancers (Hercberg et al., 2005). Cancers of the esophagus, colon, rectum, lung, and bladder are strongly associated with body iron level (Stevens et al., 1994). High iron stores, as indicated by the level of transferrin saturation exceeding 60%, increase the risks of colorectal cancer. The relative risks, adjusted for age, sex, and smoking, are 3.04 for colorectal cancer in comparison with subjects with lower iron levels (Knekt et al., 1994). Moreover, heme iron intake, serum iron, and transferrin saturation are associated with increased risks of breast cancer and death in women (Kallianpur et al., 2008; Gaur et al., 2013; Chua et al., 2016; Chang et al., 2019). High serum iron (≥120 μg/dl) is associated with elevated risks of incidence and mortality from all cancers, particularly liver and breast cancers (Wen et al., 2014). Hepcidin, encoded by the hepatic antimicrobial protein gene (*HAMP*), is a key regulator of systemic iron metabolism. It inhibits iron export by binding to FPN1, inducing its internalization and degradation. Hepcidin deficiency causes accumulation of cellular iron. The polymorphism rs10421768 in *HAMP* is reported to be associated with a three-times higher lung cancer risk (Sukiennicki et al., 2019). This polymorphism is considered to be a modulator of iron overload (Radio et al., 2015).

Some reports suggest positive correlations between iron intake and certain cancers (Beguin et al., 2014). A significant association is found between colorectal cancer risk and higher intake of heme iron and iron from red meat (Luo et al., 2019). Some endemiological studies show that people with high dietary iron intake are at a high risk for lung cancer (Kuang and Wang, 2019; Ward et al., 2019). High intake of heme iron, which is present predominantly in meat, is positively associated with breast cancer risk (Chang et al., 2019). There is a modest positive association between heme iron, total iron, and liver intakes and endometrial cancer risk (Genkinger et al., 2012).

Hereditary hemochromatosis, characterized by iron overload, is a genetic disease resulting in increased intestinal iron absorption. Patients (particularly men) with hereditary hemochromatosis are at an increased risk for hepatocellular cancer (Fracanzani et al., 2001; Elmberg et al., 2003). Blood transfusions or donation may influence the cancer risks through regulating iron stores. Donating blood removes iron from the body. Repeated blood transfusions cause iron overload. Blood donors had significantly lower mortality compared with non-donor cancer patients (Vahidnia et al., 2013). Iron reduction by phlebotomy in patients with peripheral arterial disease reduces the risk of new cancers, including lung, colorectal, upper aerodigestive, and prostate cancers (Zacharski et al., 2008). Long-term phlebotomy with low-iron diet therapy lowers the risk of development of hepatocellular carcinoma from chronic hepatitis C (Kato et al., 2007). In contrast, blood transfusion increases cancer risk (Hjalgrim et al., 2007).

### Iron Is Involved in Carcinogenesis and Tumor Growth

#### Carcinogenesis

Oxidative damage to DNA is intimately associated with carcinogenesis. Mitochondria and nicotinamide adenine dinucleotide phosphate (NADPH) oxidases (NOX) are the major sources of ROS. During the flow of electrons through the electron transport chain, some electrons leak from the electron transport chain and combine with oxygen to form superoxide. NOX transfers an electron from NADPH to molecular oxygen to generate superoxide (Panday et al., 2015). After that, the intracellular superoxide can be rapidly dismutated to hydrogen peroxide (H_2_O_2_) through superoxide dismutase (SOD). Hydrogen peroxide can be removed by the antioxidant enzyme catalase, catalyzing the conversion of hydrogen peroxide to harmless water and oxygen. If the cells have a high level of LIP, more hydroxyl radicals can be induced through Fenton reactions. Unlike superoxide and hydrogen peroxide, hydroxyl radical cannot be eliminated by antioxidant enzymes. Furthermore, hydroxyl radical has an extremely high reactivity. Hydrogen peroxide can aggressively react with any biochemical or macromolecules and causes more severe damage to the cell than any other free radicals. Hydroxyl radicals tend to cause DNA damage, resulting in the accumulation of oncogenes and mutations of tumor suppressor genes (Torti and Torti, 2020). However, direct evidence linking iron overload to carcinogenesis is still lacking.

Diseases with iron overload, such as hereditary hemochromatosis and β-thalassemia, have a high risk of liver cancer, for liver is the main site of iron storage (Fracanzani et al., 2001; Maakaron et al., 2013). Recently, Muto et al. (2019) evaluated the effects of iron overload on liver carcinogenesis. They generate a mouse model of hepatocarcinogenesis induced by hepatic iron overload, in which F-box and leucine rich repeat protein 5 (*FBLX5*) is specifically deleted in hepatocytes (Muto et al., 2019). FBLX5 is an iron–sulfur cluster protein. The oxidation state of the cluster regulates iron regulatory protein 2 (IRP2) polyubiquitination and degradation in response to both iron and oxidative stress (Wang H. et al., 2020). *FBLX5* deletion induces iron accumulation by upregulating IRP2, leading to oxidative stress, inflammation, DNA damage, liver damage, and compensatory proliferation of hepatocytes (Muto et al., 2019). These alterations consequently promote liver carcinogenesis induced by exposure to the chemical carcinogen diethylnitrosamine. *IRP2* deletion rescues the increased carcinogenesis induced by *FBLX5* deficiency (Muto et al., 2019). FBLX5–IRP2 axis is a potential therapeutic target for hepatocellular carcinoma associated with cellular iron dysregulation. It should be noted that the association between iron and carcinogenesis arises from epidemiological studies and theoretical analysis. There is limited experimental evidence regarding the role of iron in carcinogenesis. Further studies are still needed to evaluate the underlying mechanisms of how iron overload initiates and promotes carcinogenesis.

#### Tumor Growth

Iron is closely associated with cancer growth. Excess iron facilitates cancer growth, while iron deficiency caused by reduced dietary intake or iron chelators has an inhibitory effect (Torti et al., 2018). Disruption of iron homeostasis also influences tumor growth. Reduction of iron uptake through blocking TfR1 or enhancing iron efflux through overexpression of FPN1 decreases tumor growth (White et al., 1990; Pinnix et al., 2010; Deng et al., 2019). DMT1 inhibition also negatively affects the proliferation of colorectal cancer cells (Xue et al., 2016). Cancer cells require a high level of energy to support proliferation, migration, and invasion. To meet their higher demand for energy, cancer cells have an increased mitochondrial biogenesis. Complexes I, III, and IV of the mitochondrial electron transport chain contains Fe-S clusters. For this reason, mitochondria biogenesis demands cellular iron uptake. Iron may contribute to cancer progression by supporting mitochondrial electron transport chain and ATP generation. Furthermore, iron and NOX can synergistically stimulate ROS production. Iron accentuates ROS production by NOX in activated microglia. NOX2 and NOX4 inhibition significantly reduces ROS production in microglia treated with iron (Yauger et al., 2019).

### Intracellular Iron Regulation Is Altered in Cancer Cells

Generally, cancer cells require a high level of metabolically available iron by increasing iron uptake and decreasing iron storage and efflux. Manipulation of the proteins of iron metabolism may contribute to cancer growth and progression. TfR1 is highly expressed in a variety of cancers, such as leukemia, lymphoma, breast cancer, lung cancer, glioma, and others (Daniels et al., 2012). Furthermore, increased iron storage is also observed in cancer stem cells (Schonberg et al., 2015). TfR1 is a candidate marker of poor prognosis in breast cancer (Habashy et al., 2010). Therefore, TfR1 is a promising target in treating the cancers with overexpressed TfR1 and increased iron demands (Candelaria et al., 2021). Anti-TfR1 antibodies have been demonstrated to be an efficient therapy for leukemias and lymphomas (Neiveyans et al., 2019).

Serum ferritin is associated with poor prognosis in various cancers, including hepatobiliary cancer (Facciorusso et al., 2014; Song et al., 2018), lung cancer (Ji et al., 2014), pancreas cancer (Kalousová et al., 2012), T-cell lymphoma (Koyama et al., 2017), renal cancer (Singh et al., 2005) and colorectal cancer (Lee et al., 2016). Ferritin is also associated with many signaling pathways in cancer, such as p53, NF-κB (nuclear factor kappa-light-chain-enhancer of activated B cells), anti-apoptosis process, invasion, and metastasis (Alkhateeb and Connor, 2013; Min and James, 2015). Ferritin may serve as a promising and effective anticancer target. Some studies have demonstrated the therapeutic roles of ferritin in cancer treatments. Downregulation of ferritin enhances the chemosensitivity of breast cancer cells and glioma cells to chemotherapy (Liu et al., 2011; Shpyleva et al., 2011), and significantly reduces the growth rate of the tumor xenograft of melanoma cells (Di Sanzo et al., 2011).

Ferroportin 1 is the only known cellular iron efflux pump. FPN1 is downregulated in breast (Pinnix et al., 2010), prostate (Tesfay et al., 2015), and ovarian (Basuli et al., 2017) cancers. Transfection of breast cancer cells with FPN1 significantly reduces the level of intracellular iron and its growth. Increased FPN1 is associated with a cohort of breast cancer patients who have a 10-year survival rate of >90% (Pinnix et al., 2010). Overexpression of FPN1 decreases tumorigenicity and invasion of ovarian cancer cells (Basuli et al., 2017). FPN1 is tightly regulated by hepcidin, a circulating hormone mainly synthesized in the liver. However, recent studies show that hepcidin is also expressed in prostate and breast epithelial cells (Pinnix et al., 2010; Tesfay et al., 2015). Hepcidin downregulates FPN1 as an autocrine hormone, increases intracellular iron, and contributes to cancer cell progression (Pinnix et al., 2010; Tesfay et al., 2015).

These results suggest that the measurement of proteins involved in cellular iron homeostasis could be helpful in cancer prognosis. Decreasing cellular iron uptake by blocking TfR1 and increasing cellular iron export by overexpression of FPN1 are promising strategies for cancer therapy.

### Ferroptosis

Ferroptosis is a morphologically, biochemically, and genetically distinct form of regulated cell death characterized by its dependence on iron. Intracellular iron accumulation causes oxidative stress, promotes lipid peroxidation, and consequently leads to cell death (Wu et al., 2020). Compared with normal cells, several cancers strongly rely on iron to support their growth. Enhanced iron uptake and retention are considered to be the hallmarks of cancer. Since ferroptosis is a result of metabolic dysfunction involving iron and ROS, the elevated levels of iron make cancer cells more vulnerable to ferroptosis (Torti and Torti, 2020). These features suggest that ferroptosis inducers could be used to improve the efficacy of cancer therapy. Ferroptosis offers a new way of targeted cancer therapy.

**FIGURE 2:**
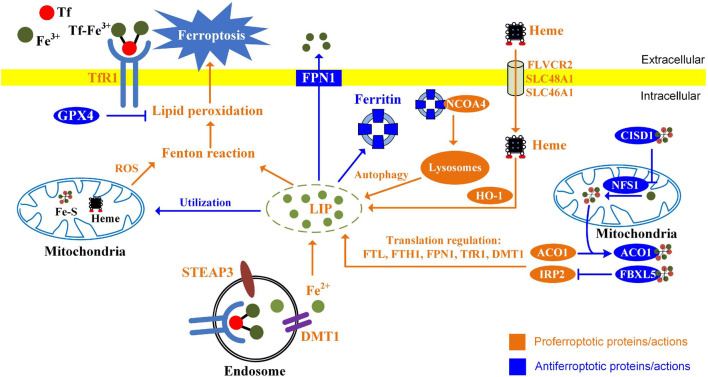
Alterations of iron metabolism influence ferroptosis in cancer cells. The ways of increasing absorption, reducing iron storage, and limiting iron efflux contribute to ferroptosis. Endocytosis of diferric Tf is the major way for cellular iron uptake. In the endosomes, ferric iron is released from Tf, reduced by STEAP3, and transported to the cytosol through DMT1. Heme provides additional sources of iron through FLVCR2, SLC48A1, and SLC46A1 in the cell membrane. HO-1 catalyzes oxidative degradation of heme to liberate ferrous iron. Ferritin is a protein that stores iron, while NCOA4-mediated ferritinophagy promotes ferritin degradation and releases ferrous iron. Iron can be utilized for the synthesis of Heme and Fe-S clusters. Mitochondrial proteins regulating iron utilization (CISD1 and NFS1) negatively regulate ferroptosis. Iron-regulatory proteins ACO1 and IRP2 regulate ferroptosis by modulating FTL, FTH1, FPN1, TfR1, and DMT1 at the translational level.

Iron accumulation and lipid peroxidation are two key factors initiating ferroptosis. Alterations of proteins involved in iron import, storage, and export have been demonstrated to influence sensitivity to ferroptosis (Figure 2; Hassannia et al., 2019). Knockdown of TfR1 suppresses ferroptosis induced by erastin or cystine deprivation (Yang and Stockwell, 2008; Gao et al., 2015). FTH1 expression levels are negatively associated with ferroptosis sensitivity (Yang and Stockwell, 2008). Knockdown of FTH1 by RNA interference enhances ferroptosis induced by erastin or sorafenib in hepatocellular carcinoma cells (Sun et al., 2016). Overexpression of PFN1 enhanced iron efflux lowers intracellular iron level and sensitizes ovarian cancer cells to ferroptosis (Basuli et al., 2017), while knockdown of ferroportin accelerates erastin (a ferroptosis inducer)-induced ferroptosis in neuroblastoma cells (Geng et al., 2018). Dysregulation of IRP cellular iron homeostasis is regulated by IRP. In iron-starved cells, IRP2 increases cellular iron levels by stabilizing TfR1 mRNA and inhibiting translation of ferritin and FPN1 (Sacco et al., 2021). Knockdown of IRP2 suppresses the sensitivity of cancer cells to ferroptosis inducers (Dixon et al., 2012). Ferritinophagy plays a pivotal role in maintaining cellular iron homeostasis by regulating the autophagic degradation of iron-storage protein ferritin (Tang et al., 2018). This process is mediated by nuclear receptor coactivator 4 (NCOA4), acting as a specific cargo receptor, binding ferritin and targeting it to emerging autophagosome (Mancias et al., 2014). Knockdown of NCOA4 inhibits iron release from ferritin. Ferritinophagy has been demonstrated to contribute to ferroptosis in cancer cells. Genetic inhibition of NCOA4 not only inhibits ferritin degradation but also suppresses ferroptosis induced by erastin or cystine deprivation (Gao M. et al., 2016; Hou et al., 2016).

Mitochondria are the center of iron utilization. In mitochondria, iron is used for the synthesis of iron–sulfur clusters and heme. Alterations of mitochondrial iron metabolism influence ferroptosis. CDGSH iron sulfur domain 1 (CISD1, also termed mitoNEET) regulates mitochondrial iron transport. Knockdown of *CISD1* is found to cause mitochondrial iron overload and to enhance erastin-induced ferroptosis (Yuan et al., 2016). Aconitase 1 (ACO1) is bifunctional. When cellular iron levels are high, it binds to iron–sulfur and catalyzes citrate to isocitrate. When cellular iron levels are low, ACO1 interacts with iron-response elements (IREs) to controls the levels of iron inside cells. Knockdown of ACO1 suppresses ferroptosis induced by cystine deprivation (Gao M. et al., 2016). NFS1 (cysteine desulfurase) is an enzyme involving synthesizing iron-sulfur clusters using sulfur from cysteine. Suppression of NFS1 cooperates with inhibition of cysteine transport to trigger ferroptosis (Alvarez et al., 2017).

It is recognized that mitochondria are one of the major sources of intracellular ROS. Mitochondrial ROS is not only important for apoptosis but also contributes to ferroptosis (Wu et al., 2021). Ferroptosis inducers, erastin or RSL3, trigger a substantial generation of mitochondrial ROS in HT-22 and MEF cells (Neitemeier et al., 2017; Jelinek et al., 2018). MitoQ, a mitochondria-targeted antioxidant, inhibits ferroptosis induced by RSL3 (Neitemeier et al., 2017). Lipid peroxidation also occurs in mitochondria during ferroptosis (Feng and Stockwell, 2018). Upon erastin treatment, lipid peroxidation first appears in mitochondria and then in plasma membrane (Gao et al., 2019). CISD1 deletion induces iron accumulation within mitochondria, and facilitates the generation of iron-mediated mitochondrial lipid peroxidation (Yuan et al., 2016). It should be noted that mitochondria play a crucial role in cysteine deprivation-induced ferroptosis but not in that induced by inhibiting GPX4 (Gao et al., 2019). Inhibition of mitochondrial tricarboxylic acid cycle (TCA cycle) or electron transfer chain (ETC) mitigates lipid peroxide accumulation and ferroptosis (Gao et al., 2019).

## Redox Balance Regulated by Nrf2 During Cancer Progression

### Nrf2-Mediated Antioxidant Response

Nrf2 is a Cap “n” Collar (CNC) basic leucine zipper (bZIP) transcription factor and is considered to be a critical regulator of cytoprotective response against oxidative and xenobiotic (or electrophilic) stresses (Bellezza et al., 2018). The Kelch-like ECH-associated protein 1 (Keap1)/Nrf2 signaling pathway plays a pivotal role in protecting against oxidative stress to maintain redox balance. Under physiological conditions, Nrf2 is constitutively maintained at a low protein level through rapid degradation *via* the Keap1-dependent proteasomal degradation. Keap1 recruits Nrf2 through binding to DLG and ETGE motifs, promotes the polyubiquitination of Nrf2 by Cullin-3 E3 ubiquitin ligase, and then induces proteasome-dependent degradation of Nrf2 by 26S proteasomal pathway. In case of oxidative stress, Nrf2 detaches from Keap1 and translocates to the nucleus, where it binds to antioxidant responsive elements (ARE) in the DNA promoter region and regulates an array of antioxidant genes, including catalase (CAT), heme oxygenase-1 (HO-1), NAD(P)H quinone dehydrogenase 1 (NQO-1), and enzymes involved in glutathione metabolism (Bellezza et al., 2018).

Nrf2-mediated antioxidant response maintains redox homeostasis and exerts anti-inflammation and anticancer activities in normal cells. However, accumulating studies suggest that Nrf2 is aberrantly activated in many cancer types, including skin, breast, prostate, and lung. Nrf2 activation is associated with poor prognosis. Nrf2 promotes cancer cell proliferation, self−renewal of cancer stem cells, anti-inflammation activities, angiogenesis, chemoresistance, and radioresistance (Wu et al., 2019). Nrf2 activation promotes aggressive lung cancer. Patients with Nrf2-activated non-squamous or squamous tumors have poor prognosis and show limited response to anti-programmed death-ligand 1 (PD-L1) treatment (Singh et al., 2021). Nrf2 activation promotes the recurrence of dormant tumor cells. Nrf2 is found to be activated in recurrent tumors in animal models and patients with breast cancer with poor prognosis. Suppression of Nrf2 impairs recurrence (Fox et al., 2020).

### Nrf2 Is Aberrantly Activated in Cancer Cells

The enhanced production of ROS is one of the fundamental features of cancer cells. Increased levels of ROS are pro-tumorigenic; supporting death evasion; uncontrolled proliferation; deregulating the cellular energetics; evading the immune response; provoking inflammation; inducing genome instability and mutations; and developing drug resistance, angiogenesis, invasiveness, and metastasis (Fouad and Aanei, 2017). Several mechanisms by which ROS production is enhanced in cancer cells have been described, such as oncogene activation, loss of cancer suppressors, and enhanced metabolism (Reczek and Chandel, 2017). Although enhanced ROS is essential for cancer survival and growth, the excess ROS must be eliminated to prevent cell death. To maintain redox balance and avoid oxidative damage, cancer cells upregulate their antioxidant capacity.

Constitutive Nrf2 activation maintains redox homeostasis by inducing the expression of antioxidants, enzymes involving GSH metabolism, and detoxification enzymes. Indeed, Nrf2 activation has been observed in various cancer types, including bladder cancer, breast cancer, cervical cancer, colon cancer, gastric cancer, globlastoma, glioma, hepatocellular carcinoma, lung cancer, multiple myeloma, pancreatic cancer, and ovarian cancer (Zimta et al., 2019). Nrf2 activation contributes to tumor growth, metastasis, and resistance to chemotherapy (Zimta et al., 2019). Constitutive activation of Nrf2 accelerates the recurrence of dormant tumor cells following therapy through regulation of redox and nucleotide metabolism (Fox et al., 2020). Furthermore, Nrf2 activation promotes NADPH production by regulating the pentose phosphate pathway and serine biosynthesis pathways (Wu et al., 2011; DeNicola et al., 2015). Hypoxia arises in tumor regions due to inadequate oxygen delivery, when the tumor rapidly outgrows its blood supply (Toth and Warfel, 2017). Hypoxia is well known to increase ROS production, eliciting oxidative stress. In response to hypoxia, Nrf2-mediated antioxidant pathway is also activated to improve hypoxia adaptation and cancer pathogenesis (Shi et al., 2019).

Several studies have demonstrated the underlying mechanisms of the constitutive activation of Nrf2 in cancers. Oncogenes, K-Ras, B-Raf, and Myc each increased the transcription of Nrf2 and lower intracellular ROS (DeNicola et al., 2011). Loss of KEAP1 interaction domain by missing exon 2, or exons 2, and 3 in *NFE2L2* gene (encoding Nrf2), results in the failure of interaction with Keap1 (Goldstein et al., 2016). Succination of Keap1 cysteine residues abrogates its binding affinity with Nrf2 (Adam et al., 2011). Somatic mutations in Nrf2, CUL3, and SIRT1 confer an Nrf2 activation phenotype in cancer cells (Ooi et al., 2013). P1 region, including 12 CpG sites, is highly methylated in the Keap1 promoter, resulting in Keap1 downregulation (Wang et al., 2008). p62 interacts with the Nrf2-binding site on Keap1, leading to stabilization of Nrf2 (Komatsu et al., 2010). The KRR motif in p21 directly interacts with the DLG and ETGE motifs in Nrf2 and thus competes with Keap1 for Nrf2 binding, compromising the ubiquitination of Nrf2 (Chen et al., 2009).

### Nrf2 Is a Potential Target for Cancer Therapy

Induction of ROS and oxidative stress is suggested to be involved in cancer initiation and progression. Cancer cells exhibit higher levels of ROS than normal cells (Hayes et al., 2020). The large amount of ROS activates Nrf2, preventing the possible oxidative damage induced by excess ROS. Nrf2 plays an important role in cancer progression, therapy resistance, and poor prognosis (Panieri and Saso, 2019). Therefore, Nrf2 is a potential therapeutic target in cancer therapy. Some Nrf2 inhibitors have been found with anti-cancer efficacy by suppressing Nrf2 expression, causing Nrf2 degradation or inhibiting Nrf2 nuclear translocation (Qin et al., 2019). These Nrf2 inhibitors include natural compounds derived from medicinal plants and some commercial drugs, such as procyanidin (Ohnuma et al., 2015, 2017), flavonoid luteolin (Chian et al., 2014), alkaloid trigonelline (Arlt et al., 2013), quassinoid brusatol (Tao et al., 2014; Olayanju et al., 2015; Karathedath et al., 2017; Wang M. et al., 2018), chrysin (Wang J. et al., 2018), oridonin (Lu et al., 2018), convallatoxin (Lee et al., 2018), honokiol (Gao D. Q. et al., 2016), wogonin (Qian et al., 2014; Xu et al., 2014, 2017; Kim et al., 2016), etc. Moreover, some commercial drugs, such as ascorbic acid (Tarumoto et al., 2004), retinoic acid (Valenzuela et al., 2014), antitubercular agent isoniazid (Verma et al., 2018), CDK inhibitor PHA-767491 (Liu et al., 2018), sorafenib (Zhou et al., 2013), valproic acid (Cha et al., 2016), metformin (Do et al., 2013, 2014; Yu et al., 2017; Sena et al., 2018), and glucocorticoid clobetasol propionate (Choi et al., 2017) also have anti-Nrf2 activity (Jung et al., 2018; Panieri and Saso, 2019). These Nrf2 inhibitors are summarized in Table 1.

**TABLE 1:** Nrf2 inhibitors in cancer research.

**Compounds**	**Types of cancer/cell lines**	**Main findings**	**References**
Procyanidin	Non-small-cell lung cancer/A549.	Reduce Nrf2 expression, and promote proteasome-independent degradation of nuclear Nrf2.	Ohnuma et al., 2015, 2017;
Luteolin	Non-small-cell lung cancer/A549.	Reduce Nrf2 expression and its downstream antioxidant enzymes.	Chian et al., 2014
Trigonelline	Pancreatic carcinoma/Panc1, Colo357, and MiaPaca2.	Block Nrf2-dependent proteasome activity.	Arlt et al., 2013
Brusatol	Acute myeloid leukemia/TPH1; Non-small-cell lung cancer/A549; Hepatoma/Hepa-1c1c7; Pancreatic cancer/PATU-8988 and PANC-1; Melanoma/A375.	Suppress Nrf2 pathway.	Tao et al., 2014; Olayanju et al., 2015; Karathedath et al., 2017; Wang M. et al., 2018
Chrysin	Glioblastoma/T98, U251, and U87.	Inhibit ERK/Nrf2 pathway.	Wang J. et al., 2018
Oridonin	Osteosarcoma/MG63 and HOS.	Suppress Nrf2 mediated antioxidant pathways.	Lu et al., 2018
Convallatoxin	Non-small-cell lung cancer/A549.	Suppression of Nrf2 is regulated at the level of proteolysis.	Lee et al., 2018
Honokiol	Lymphoid cancer/Raji and Molt4	Attenuate Nrf2 and NF-κB.	Gao D. Q. et al., 2016
Wogonin	Hepatoma/HepG2; Head and neck cancer/HNC; Leukemia/K562.	Inhibit Nrf2 *via* Stat3/NF-κB signaling.	Qian et al., 2014; Xu et al., 2014; Kim et al., 2016; Xu et al., 2017
Ascorbic acid	Leukemia/Imatinib-resistant KCL22.	Inhibit Nrf2 nuclear translocation.	Tarumoto et al., 2004
Retinoic acid	Acute myeloid leukemia/HL60.	Inhibit Nrf2 nuclear translocation.	Valenzuela et al., 2014
Isoniazid	Hepatoma/HepG2.	Inhibit Nrf2 nuclear translocation.	Verma et al., 2018
PHA-767491	Hepatoma/HepG2; Myeloma/MM.1S, L363, and U266.	Inhibit Nrf2 nuclear translocation.	Liu et al., 2018
Sorafenib	Hepatoma/5-FU resistant Bel-7402.	Suppress Nrf2 expression.	Zhou et al., 2013
Valproic acid	Papillary thyroid cancer/TPC1 and BCPAP.	Inhibit Nrf2 nuclear expression.	Cha et al., 2016
Metformin	Hepatoma/HepG2; Breast cancer/MCF-7; Colon cancer/HT29; Non-small-cell lung cancer/A549, H1299, and H460.	Suppress Nrf2 expression *via* downregulating PPARγ transcriptional activity.	Do et al., 2013; Do et al., 2014; Yu et al., 2017; Sena et al., 2018
Clobetasol propionate	Non-small-cell lung cancer/A549.	Suppress Nrf2 nuclear translocation and promote its degradation.	Choi et al., 2017

## The Regulatory Role of Nrf2 in Iron Homeostasis

Free iron is toxic to the body by catalyzing the production of highly reactive hydroxyl radicals (OH⋅) through Fenton reaction. Therefore, the intracellular LIP must be tightly regulated. To date, most of the current researches on Nrf2 focus on its antioxidant properties. However, Nrf2 has been demonstrated to play a pivotal role in iron homeostasis. Nrf2 can regulate iron storage and iron efflux through ferritin, FPN1, and HO-1 gene transcription (Kasai et al., 2018).

### Cellular Iron Homeostasis

Ferritin is a highly conserved and universal iron storage protein composed of 24 subunits of two types, ferritin heavy chain (FTH) and ferritin light chain (FTL). Ferritin can carry up to 4,500 iron atoms in its core and prevents iron-mediated formation of harmful ROS (Arosio et al., 2017). Therefore, ferritin has an antioxidant effect to some extent. The effects of Nrf2 on iron storage are first found in Nrf2-deficient mice. The *Nrf2*^–/–^ mice exhibit abnormally white teeth due to defective iron utilization during the development of tooth enamel (Yanagawa et al., 2004). Subsequent research shows Nrf2 activation is responsible for the induction of ferritin. Some chemo-preventive agents, such as sulforaphane and 1,2-dithiole-3-thione, can activate Nrf2 and induce ferritin transcription (Kwak et al., 2001; Thimmulappa et al., 2002). Electrophoretic mobility shift assays demonstrate that Nrf2 binds ARE elements and mediates the transcriptional activation of ferritin (Pietsch et al., 2003). The induction of ferritin is not observed in Nrf2 knockout cells. These results suggest that Nrf2 mediates the induction of ferritin H and L in response to xenobiotics (Pietsch et al., 2003).

Nrf2 not only regulates iron homeostasis by inducing ferritin transcription but also decreases LIP by enhancing iron efflux of the cell. FPN1 is the only known mammalian iron exporter of non-heme iron. Nrf2 activation promotes FPN1 mRNA expression and enhances iron release (Harada et al., 2011). The ARE element located at position -7007/-7016 of *FPN1* promoter is involved in Nrf2-mediated transcription (Marro et al., 2010). The induction of *FPN1* by Nrf2 can be also regulated by BTB and CNC homology 1 (Bach1). Bach1 and Nrf2 compete to bind ARE-like enhancers in cells and regulate ARE-mediated FPN1 expression. The role of Bach1 in Nrf2-mediated *FPN1* regulation arises from studies on the heme induced *FPN1* expression in macrophages. Before erythrophagocytosis or Heme treatment, Bach1 binds to the ARE of *FPN1*. Upon Heme treatment, the heme-sensitive Bach1 releases from DNA and allows Nrf2 to bind, consequently promoting *FPN1* transcription (Marro et al., 2010). Whether Bach1 inactivation is involved in the regulation of ferritin transcription needs further investigation.

Heme, an iron-containing molecule, plays an essential role in mitochondrial electron transport as a cofactor of cytochromes. Heme can enter cell through FLVCR2 (feline leukemia virus subgroup C receptor-related protein 2), SLC48A1, and SCL46A1 in the cell membrane (Cao and Fleming, 2015). Its degradation is regulated by HO-1, which is also an Nrf2-regulated gene. HO-1 (encoded by *HMOX1*) is an initial and rate-limiting enzyme catalyzing the oxidative degradation of heme to produce biliverdin, ferrous iron, and carbon monoxide. Biliverdin is subsequently converted to bilirubin by biliverdin reductase (Chau, 2015). HO-1 overexpression may lead to the accumulation of iron derived from HO-1 catabolism and mediates the development of ferroptosis induced by erastin, withaferin A, and BAY 11-7058 (Kwon et al., 2015; Chang et al., 2018; Hassannia et al., 2018). Pharmacological inhibition or knockdown of HO-1 has an inhibitory effect (Kwon et al., 2015; Chang et al., 2018; Hassannia et al., 2018). It has been proven that Nrf2/HO-1 mediates tagitinin C-induced ferroptosis (Wei R. et al., 2021). In addition to its primary role in heme catabolism, HO-1 exhibits anti-oxidative functions *via* the actions of bilirubin (Chau, 2015). Activation of Nrf2/HO-1 axis is also involved in the cytoprotective effects against ferroptosis (Jiang et al., 2020; Ma et al., 2020; Wang et al., 2021; Wei N. et al., 2021). Whether HO-1 is cytoprotective or cytotoxic, it may depend on the degree of its expression. Excessive upregulation of HO-1 releases a significant amount of ferrous iron from heme and enhances the generation of ROS through Fenton reaction, while a moderate upregulation may be cytoprotective (Hassannia et al., 2019).

### Systemic Iron Homeostasis

Nrf2 deficiency dysregulates iron homeostasis in the body. Nrf2 deficiency aging mice exhibit an increased accumulation of iron in liver, spleen, and serum. Iron regulatory genes responsible for uptake (TfR1 and DMT1) and excretion (FPN1) are decreased, while ferritin responsible for iron deposition (FTL and FTH1) is upregulated (Liu et al., 2020). If Nrf2 is responsible for the dysregulation of iron homeostasis, the ferritin should be decreased. However, Nrf2 knockout enhances ferritin expression in aging mice. These results suggest that ferritin, FPN1, DMT1, and TfR1 may be regulated by IRP-iron responsive element (IRE) system in Nrf2 deficiency mice. Nrf2 controls systemic iron homeostasis *via* BMP6/hepcidin in hepatocytes (Lim et al., 2019). Nrf2 regulates Bmp6 expression and is required for the induction of hepatic hepcidin in response to mitochondrial ROS and oxidative damage mediated by iron. Nrf2-deficient mice with iron overload exhibit defective hepcidin induction (Lim et al., 2019). Pharmacological Nrf2 activation not only improves iron homeostasis in hemochromatosis and thalassemia but also induces antioxidant/detoxifying enzyme gene expression to alleviate iron-mediated oxidative damage (Lim et al., 2019).

## Nrf2 Is a Potential Modulator Orchestrating Iron Homeostasis and Redox Balance in Cancer Cells

Cancer cells have extremely higher energy demands, which provides a fundamental advantage for proliferation, migration, and metastasis. Therefore, the reliance on mitochondrial energy generation is much greater than normal cells. To meet their high energy demand, cancer cells have an enhanced mitochondrial biogenesis and possess abundant mitochondria (Zong et al., 2016). Iron is an essential trace element for mitochondrial biogenesis. Mitochondria contain up to 20–50% of total cellular iron (Ward and Cloonan, 2019). Mitochondrial iron is utilized for the biosynthesis of Fe-S clusters involving in electron transport. Alterations in mitochondrial iron concentrations can impair the biosynthesis of Fe-S clusters, leading to mitochondrial dysfunction, and increase oxidative stress. Iron depletion initiates a rapid and reversible decrease in mitochondrial biogenesis through dampening the transcription of genes encoding mitochondrial proteins (Rensvold et al., 2016). Therefore, cancer cells require abundant iron uptake to support tumor growth and migration. However, excess iron may generate a large abundant of ROS through Fenton reaction and induce oxidative stress and even damage to the cells. As the major source of ROS, 90% intracellular ROS is demonstrated to be generated in mitochondria. Since the cancer cells have a large number of mitochondria, cancer cells overproduce about a 10-fold level of ROS compared with normal cells (Zhang et al., 2019). How the cancer cells perceive the intracellular iron concentration, regulate iron uptake, and stimulate antioxidant system is of great importance.

**FIGURE 3:**
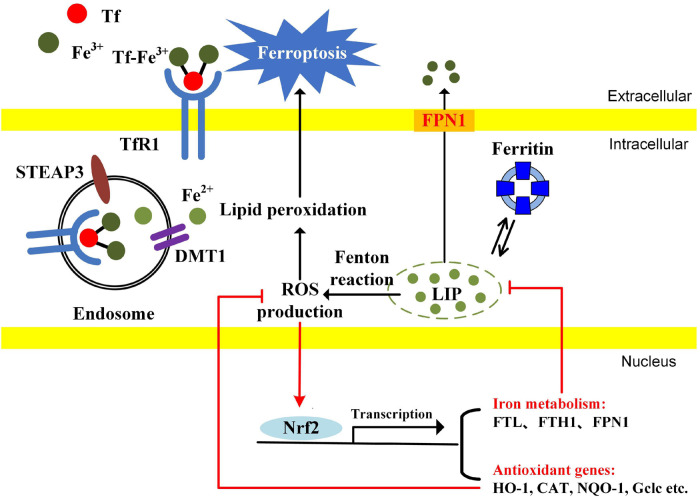
The possible mechanisms of Nrf2 modulating iron metabolism and redox balance in cancer cells. The cancer cells have an enhanced iron uptake compared with normal cells. The LIP, a free cellular iron pool, generates ROS through Fenton reaction, causing the activation of Nrf2. Nrf2 translocates into the nucleus and promotes the transcription of genes associated with antioxidant enzymes and iron metabolism. Ferritin and FPN1 reduce the cellular free iron. Antioxidant enzymes reduce the ROS accumulation. Since cellular free iron and iron-dependent lipid peroxidation are essential for ferroptosis induction, Nrf2 inhibition probably contributes to ferroptosis by simultaneously enhancing LIP and oxidative stress.

Nrf2 is a potential modulator capable of orchestrating iron homeostasis and redox balance. Nrf2 is first identified to have redox-regulating capacities by recognizing ARE for transcription activation of antioxidant genes, including HO-1, CAT, NQO-1, glutamate-cysteine ligase catalytic subunit (Gclc), etc. Recent studies have revealed that Nrf2 is involved in iron homeostasis. *FPN1* (iron efflux proteins) and *ferritin* (including *FTH1* and *FTL*) are the new target genes of Nrf2. Iron-mediated ROS production activates Nrf2 and causes Nrf2 nuclear translocation. Nrf2 not only induces the gene expression of anti-oxidant enzymes but also gene expression involved in iron metabolism. FPN1 controls iron export from cells. The iron storage protein ferritin contributes to iron storage. Both FPN1 and ferritin reduce the intracellular free iron and limit the production of iron-mediated ROS (Figure 3). Nrf2 is a potential modulator for orchestrating iron homeostasis and redox balance in cancer cells.

## The Potential Application

In view of the constitutive activation of Nrf2 in various cancers, Nrf2 is believed to be a potential therapeutic target. Therefore, inhibition of the Nrf2 pathway is a useful strategy for cancer therapy and reversing drug resistance. Some Nrf2 inhibitors have been identified and reported to have anti-cancer activities. However, most of these Nrf2 inhibitors exhibit low potency, non-specificity, and inconsistency. There have been no inhibitors currently and clinically available or under clinical trial (Jung et al., 2018; Panieri and Saso, 2019).

Increased iron stores are associated with cancer induction, malignant progression, therapy resistance, and immune evasion. The enhanced iron demand makes cancer cells more vulnerable to iron-dependent ferroptosis. However, excess iron induces ROS production *via* Fenton reaction, activating Nrf2 mediated antioxidant pathways to avoid iron-induced oxidative damage. Since ferroptosis is strongly dependent on antioxidant metabolism and iron homeostasis, genes or pathways associated with metabolism in iron or antioxidative stress may potentially regulate sensitivity to ferroptosis. Nrf2 regulates genes involving not only antioxidant defenses but also the synthesis of heme and Fe-S clusters, iron storage, and iron export. Nrf2 is a core regulator orchestrating the activation of antioxidant defenses to protect against iron toxicity. If cancer cells are treated with ferroptosis inducers along with Nrf2 inhibitors, it may effectively increase the sensitivity to ferroptosis. Indeed, Nrf2 plays a central role in protecting against ferroptosis by activating transcription of NQO-1, HO-1, and FTH1. Inhibition of Nrf2 or its downstream antioxidant genes and FTH1 promotes ferroptosis in response to ferroptosis-inducing compounds (erastin and sorafenib) (Sun et al., 2016). Nrf2 inhibition reverses the resistance of cisplatin-resistant head and neck cancer cells to artesunate-induced ferroptosis (Roh et al., 2017). Nrf2 inhibition also reverses resistance to GPX4 inhibitor-induced ferroptosis in head and neck cancer (Shin et al., 2018). Nrf2 inhibitors are the promising modulators of ferroptosis.

## Conclusion

In this hypothesis, we highlight the role of Nrf2 in ferroptosis through regulating iron and ROS homeostasis. Nrf2 plays dual roles in both iron homeostasis and redox balance. Iron promotes carcinogenesis, tumor growth, and migration. On the other hand, excess iron also causes overproduction of ROS through Fenton reaction and mitochondrial oxidative respiration. To avoid the excess of redox-active iron and oxidative damage, it is highly probable that Nrf2 modulates the balance between iron status and oxidative stress. Nrf2 can be activated by iron-induced ROS and induces transcriptions of antioxidant genes, FPN1, and ferritin. Hence, Nrf2 not only reduces cellular oxidative stress but also decreases the free iron level. Since ferroptosis is dependent on lipid peroxide and iron accumulation, Nrf2 inhibition may dramatically increase the sensitivity to ferroptosis. The combination of Nrf2 inhibitors with ferroptosis inducers may exert greater efficacy on cancer therapy.

## Data Availability Statement

The original contributions presented in the study are included in the article/supplementary material, further inquiries can be directed to the corresponding author/s.

## Author Contributions

JZ contributed to the study conception and design and wrote the manuscript. LZ conducted the literature search. YJ drew the figures. GY, HZ, PQ, and SW revised the manuscript. All authors contributed to the article and approved the submitted version.

## Conflict of Interest

The authors declare that the research was conducted in the absence of any commercial or financial relationships that could be construed as a potential conflict of interest.

## Publisher’s Note

All claims expressed in this article are solely those of the authors and do not necessarily represent those of their affiliated organizations, or those of the publisher, the editors and the reviewers. Any product that may be evaluated in this article, or claim that may be made by its manufacturer, is not guaranteed or endorsed by the publisher.
